# Supplementary material for: Dynamic transcriptomic profiles of zebrafish gills in response to zinc supplementation
Source: BMC Genomics. 2010 Oct 11;11:553. doi: 10.1186/1471-2164-11-553 (PMC3091702; doi:10.1186/1471-2164-11-553)
Supplement: Additional file 2 — Interactive Direct Interaction Network representing the molecular interactions between zinc, copper, iron, calcium and proteins encoded by transcripts changed by zinc supplementation. Mini web-site containing index.html and hyperlinked pages in subdirectory describing a Direct Interaction Network automatically generated based on curated interactions contained within the proprietary PathwayArchitect database. Ovals represent proteins and the circles symbolize metal ions. Objects are coloured by their abundance in zebrafish at the time-point they were significantly different from the control is a scale from -4 fold (dark green) to +4 fold (dark red). Where significant differences were found at more than one time-point, the colour overlay shows expression at the first instance. Dark blue squares denote 'binding', and light blue squares 'expression'; green squares stand for 'regulation', green diamonds for 'metabolism', and green circles for 'promoter binding'. Arrow heads indicate directionality of the interaction where annotated. All nodes and edges can be further interrogated by selecting the relative area of the image. [file 1471-2164-11-553-S2.zip › PathwayArchitect Zn xs DIN/1593173.html]

# BINDING:

|  |  |
| --- | --- |
| Type | BINDING |
| Effect | None |


---

|  |  |
| --- | --- |
| Score | 0 |


---

|  |  |
| --- | --- |
| Reference Count | 1 |


---

|  |  |
| --- | --- |
| Mechanism | Unknown |


---

|  |  |
| --- | --- |
| Reference:0 || PMID | 15604093 |
| SourceID | 215745 |
| Species | Human |
| Experimental Condition | in-vivo |
| Description | An interaction between NR1I2 and RXRbeta was demonstrated by yeast two hybrid screen. CG1945 was transformed with NR1I2, bait, fused to the GAL4 DNA binding domain (DBD) and mated with Y187 containing a human liver cDNA library which included RXRbeta[164-533], prey, fused to the GAL4 activation domain (AD). Interactions were detected by growth on media lacking histidine and containing 3-aminotriazole and by beta-galactosidase activity, in the presence of 0.4 um of the ligand rifampicin. Interacting proteins were identified by DNA purification and sequencing of positive clones. High confidence subsets of interaction pairs were those that matched 2 or 3 out of 3 criteria: multiple independent clones isolated, statistic parameters, and interaction pattern. Supplemental data 1. |
| Detection Method | two-hybrid-test |
| Source | BIND |
  |


---

|  |  |
| --- | --- |
